# Probiotics Supplementation in Tuberculosis: A Scoping Review

**DOI:** 10.1155/sci5/6926727

**Published:** 2025-11-10

**Authors:** Tejaswini Baral, Mohan K. Manu, Kavitha Saravu, Chandrashekar Udyavara Kudru, Jitendra Singh, Chiranjay Mukhopadhyay, Mahadev Rao, Sonal Sekhar Miraj

**Affiliations:** ^1^Department of Pharmacy Practice, Manipal College of Pharmaceutical Sciences, Manipal Academy of Higher Education, Manipal 576104, Karnataka, India; ^2^Department of Respiratory Medicine, Kasturba Medical College, Manipal, Manipal Academy of Higher Education, Manipal 576104, Karnataka, India; ^3^Department of Infectious Diseases, Kasturba Medical College, Manipal, Manipal Academy of Higher Education, Manipal 576104, Karnataka, India; ^4^Department of Medicine, Kasturba Medical College, Manipal, Manipal Academy of Higher Education, Manipal 576104, Karnataka, India; ^5^Department of Translational Medicine, All India Institute of Medical Sciences, Bhopal 462020, Madhya Pradesh, India; ^6^Department of Microbiology, Kasturba Medical College, Manipal, Manipal Academy of Higher Education, Manipal 576104, Karnataka, India

**Keywords:** antitubercular therapy, probiotics, tuberculosis

## Abstract

This scoping review aims to synthesize the current clinical evidence on probiotics used in tuberculosis (TB). Systematic literature searches were conducted across PubMed, Scopus, and Embase databases to identify all studies using probiotics in TB. A total of six studies conducted between 2016 and 2023 were found and included in this review. All the studies incorporated probiotics supplementation not beyond the intensive phase of antitubercular therapy (ATT), ranging from three to eight weeks. Five out of six included studies focused on pulmonary TB. Probiotics alleviate adverse gastrointestinal and hepatic drug reactions, modulate gut microbiota, enhance barrier function, and influence immune responses. Therefore, probiotics are a potential adjunct therapy during the intensive phase of ATT. However, their long-term effects remain unexplored, highlighting the future research scope for well-designed longitudinal studies to explore their sustained benefits.

## 1. Introduction

Tuberculosis (TB) is a chronic infectious illness caused by *Mycobacterium tuberculosis*. It remains a significant global public health concern, ranking among the foremost causes of mortality from infectious diseases globally [1]. In 2023, the incidence of TB was 10.8 million, according to the Global TB Report 2024. In 2023, 8.2 million individuals worldwide were newly diagnosed with TB, a rise from 7.5 million in 2022 [1]. Curative treatment for TB is available, but it requires long-term drug therapy and may expose patients to drug-induced adverse drug reactions (ADRs). The standard recommended treatment for drug-sensitive TB is a 2-month intensive phase, followed by a 4-month continuation phase of combination anti-TB drugs [1]. In other cases of TB, like mono or multidrug resistance, the treatment duration lasts up to 24 months [1]. Scientific evidence [2] suggests that TB disease and its treatment disturb gut microbiota diversity. The microbial balance can be significantly changed by antitubercular therapy (ATT) and the infection itself. These disruptions might adversely affect patient health and treatment outcomes [2]. Probiotics are defined by the WHO and the Food and Agriculture Organization as “live microorganisms which, when administered in adequate amounts, confer a health benefit on the host” [3].

Externally administered probiotics containing specific species or strains can temporarily colonize the intestinal tract and restore microflora stability. They help restore essential physiological functions and promote commensal flora [4]. Considerable human health problems are managed with probiotics, including irritable bowel syndrome, *Helicobacter pylori* infection, inflammatory bowel disease, antibiotic-induced diarrhea, and constipation [5]. Researchers are increasingly investigating probiotics for their potential role in managing cancer, cardiovascular, cerebrovascular, and infectious diseases [5]. Furthermore, emerging evidence indicates that probiotics may confer advantageous effects in infectious diseases, including pneumonia, and influenza [6, 7]. Considering the emerging evidence regarding the role of probiotics in modulating gut microbiota and their potential immunomodulatory advantages, there is increasing interest in investigating their use as an adjunctive strategy in TB management [8]. Nonetheless, the existing evidence regarding the application of probiotics in TB patients is fragmented and inadequately synthesized. This scoping review aims to systematically synthesize the current clinical evidence on probiotics use in TB.

## 2. Methods

The scoping review complies with the PRISMA extension for scoping reviews (PRISMA-ScR) checklist (available here) [9]. The PICOS [10] format we followed is as follows: Population-Patients with TB; Intervention/Exposure-Any kind of probiotics supplementation; Comparison-Not applicable; Outcome-Efficacy; and Study design-All types.

### 2.1. Eligibility Criteria and Information Sources

This review incorporated clinical studies exploring probiotic supplementation's effects in TB. Any reviews or preclinical studies were excluded. Studies that were published in languages other than English were translated into English.

A systematic search was conducted using PubMed, Scopus, and Embase databases to identify eligible research studies. A manual search was also performed to ensure thorough coverage of relevant literature. The search was conducted from inception to February 2025, and no publication date restrictions were applied. The key search terms included “tuberculosis” and “probiotics,” along with their synonyms and medical subject headings (MeSH) terms. These terms were combined using Boolean operators (AND, OR) to enhance the sensitivity and precision of the search strategy.

### 2.2. Selection of Sources of Evidence and Data-Charting Process

The identified articles were subjected to title and abstract screening followed by full-text screening according to our study eligibility criteria. One reviewer (T.B.) performed the search, screening, and data extraction, which was checked by another reviewer (S.S.M.). We resolved disagreements on study selection and data extraction by consensus and discussion with other reviewers if needed. A data-charting form using Microsoft Excel was developed by one reviewer (T.B.) to determine which variables to extract. Two reviewers (T.B. and S.S.M.) discussed the results and continuously updated the data-charting form in an iterative process.

### 2.3. Data Items and Synthesis of Results

We extracted the data on the following characteristics: title, country, study design, study duration, type of probiotics, the composition of probiotics, study population, any other exposure/intervention with probiotics supplementation, and study outcome. Details of the data items are provided in the supplementary file. We performed a descriptive synthesis to map the available evidence on the impact of probiotic supplementation in TB. The primary study outcomes from individual studies were considered during the synthesis to provide a comprehensive overview of the existing literature. Due to the heterogeneity in outcome measures across the included studies, a quantitative synthesis was not feasible. Therefore, the results are presented using a narrative summary, organized thematically according to the outcome assessed. Given the nature of this scoping review, no formal risk of bias assessment was conducted.

## 3. Results

### 3.1. Selection of Sources of Evidence

**Figure 1:**
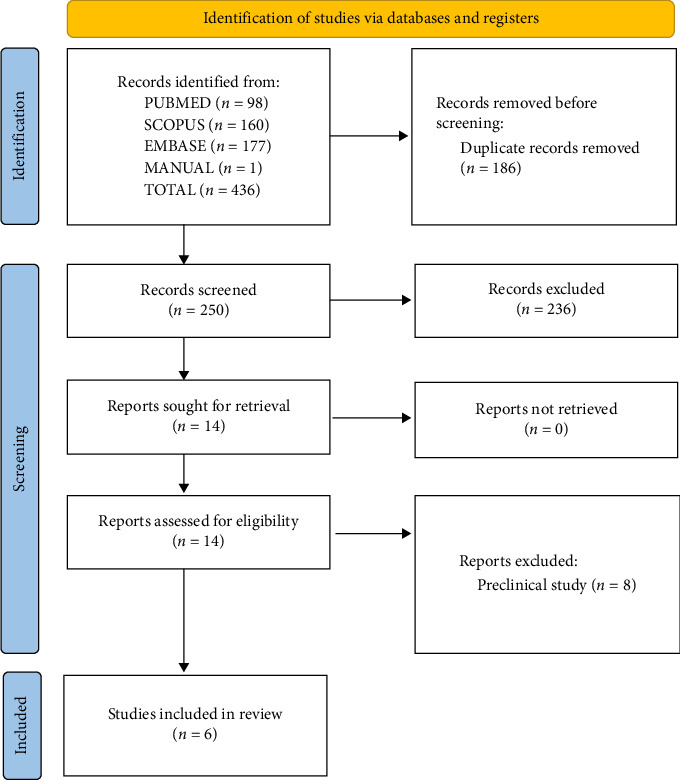
PRISMA flowchart.

A total of 436 records were identified from the abovementioned bibliographic databases and a manual search. We screened 250 records from the abstract title after removing 186 duplicates. Among those, 236 records were excluded because they were irrelevant to our review. Finally, 14 studies were subjected to full text screening, of which eight were excluded because they were preclinical studies. Finally, six studies [11–16] were included in our review (Figure 1).

### 3.2. Characteristics of Included Studies

All six included studies were published between 2016 and 2023 and were reported from Russia, China, and Indonesia; this reflects the fact that these are the only clinical investigations on probiotics in TB identified through our comprehensive search [11–16]. The details of the study designs of the included studies are mentioned in Table 1. Among the six included studies, two studies, i.e., Xiong et al. [12] and Jiang et al. [13] were post hoc analyses of a single RCT, i.e., Lin et al. [14]. However, they focused on different outcomes related to probiotics supplementation in TB patients [12–14].

Five of the six studies were conducted on patients with drug-sensitive pulmonary TB (PTB) [12–15], while one was on multidrug-resistant pulmonary TB (MDR-PTB) [11]. One study included patients with extrapulmonary TB (EPTB) in addition to PTB [15].

### 3.3. Probiotics Supplementation Characteristics

The composition of probiotics used across the included studies varied considerably. Otdushkina et al. [11] and Suprapti et al. [15] used multistrain probiotics formulations combining different species of *Lactobacillus*, *Bifidobacterium*, and *Streptococcus*. Three studies, i.e., Lin et al. [14], Xiong et al. [12], and Jiang et al. [13] used *Lactobacillus casei* as the probiotic strain, all derived from the same randomized controlled trial conducted. Setiyaning et al. [16] did not report the specific probiotic strains used. The duration of probiotics supplementation among the included studies ranged from 3 weeks to 8 weeks (Table 1).

Two studies combined probiotics with micronutrient supplementation [15, 16]. Suprapti et al. [15] combined probiotics with vitamins B1, B6, and B12. Setiyaning et al. [16] combined probiotics with zinc supplementation.

### 3.4. Outcome Measures

**Figure 2:**
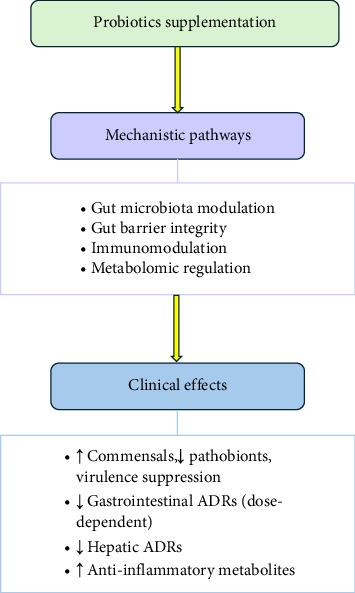
Summary of the proposed mechanisms and clinical effects of probiotic supplements in TB from included clinical studies.

The included studies assessed a wide range of outcomes related to probiotics supplementation in TB patients (Table 2). A schematic summary of proposed mechanisms and clinical effects reported across these studies [11–16] is presented in Figure 2.

#### 3.4.1. Incidence of ATT-Induced Gastrointestinal (GI) ADRs

Lin et al. [14] studied the impact of probiotic supplementation on the incidence of ATT-induced GI ADRs. The study demonstrated that administering *Lactobacillus casei*, especially in higher doses of 2 × 10^10^ CFU, two bottles of 100 mL per day, reduced both the incidence and duration of GI ADR associated with ATT compared with individuals who did not receive *Lactobacillus casei.* The nonprobiotic group reported a higher incidence of issues such as vomiting, anorexia, and constipation [14].

#### 3.4.2. Incidence of ATT-Induced Liver Injury and Hepatological Parameters

Xiong et al. [12] reported the impact of probiotics supplementation on the incidence of ATT-induced liver injury and hepatological parameters. Xiong et al. [12] reported that, while the overall incidence of liver injury during ATT did not significantly differ between groups, there was a declining trend associated with probiotic use, decreasing from 5.7% in the nonprobiotic group to 3.5% in the high dose (2 × 10^10^ CFU, two bottles of 100 mL per day) *Lactobacillus casei* group. The high-dose group exhibited a significantly reduced incidence of elevated alkaline phosphatase (ALP), while the low-dose group demonstrated a markedly lower prevalence of elevated total bilirubin.

#### 3.4.3. Gut Microbiota Composition

Otdushkina et al. [11] and Xiong et al. [12] studied the gut microbiota composition. Otdushkina et al. [11] studied gut microbiota using a culture-dependent method, whereas Xiong et al. [12] used the culture-independent high-throughput sequencing-based analysis of the microbiome using 16S V3-V4 sequencing.

In the study by Xiong et al. [12], both low-dose (1 × 10^10^ CFU, one bottles of 100 mL per day) and high-dose (2 × 10^10^ CFU, two bottles of 100 mL per day) probiotic groups exhibited significant shifts in gut microbial community compared with the control as evidenced by beta diversity indices. However, no significant difference was observed between the low- and high-dose groups regarding diversity. At the taxonomic level, both probiotic groups showed a marked decrease in *Bacteroidetes* and an increase in *Actinobacteria*. The marker taxa in Xiong et al. [12] revealed an increased abundance of potentially beneficial genera like *Bifidobacterium*, *Blautia*, *Streptococcus*, and *Collinsella* in low- and high-dose probiotic groups [12].

In the study by Otdushkina et al. [11], post probiotic supplementation, the following are concluded: (1) increase in beneficial commensals, (2) reduction in pathogens and opportunistic microorganisms, (3) virulence suppression, and (4) improvement in functional profile of isolates. Before probiotics supplementation, Otdushkina et al. [11] found a significant dysbiosis characterized by reduced levels of beneficial bacteria like *Bifidobacterium*, *Lactobacillus*, *Escherichia coli lac*+, and increased presence of opportunistic and potentially pathogenic microorganisms, like *Staphylococcus spp.*, *Candida* fungi, *Clostridium perfringens*, and lactose-negative *Escherichia coli*. However, postsupplementation, Otdushkina et al. [11] observed a significant increase in *Lactobacillus* counts, reduction in the prevalence and titers of *Candida* fungi, lactose-negative *E. coli*, and hemolysin-producing *Staphylococcus* and *Enterococcus* strains. The activity of virulence factors such as lipase and protease enzymes were significantly reduced after supplementation. For functional trait analysis, Otdushkina et al. [11] conducted a fatty acid composition analysis of *Enterococcus* cell membranes, revealing posttreatment structural changes indicative of altered membrane integrity and metabolic function. Additionally, a significant increase in organic acid production during carbohydrate fermentation by *Enterococcus faecalis* and *Enterococcus faecium* was observed, suggesting enhanced biochemical functionality [11].

#### 3.4.4. Intestinal Permeability

Xiong et al. [12] reported the impact of probiotics supplementation on intestinal permeability parameters. They found a significant decline in plasma levels of lipopolysaccharide (LPS), zonula occludens-1 (ZO-1), and intestinal fatty acid binding protein (I-FABP) with high-dose probiotics supplementation, which implies change in intestinal permeability and a drop in bacterial translocation.

#### 3.4.5. Immune/Inflammatory Markers

Jiang et al. [13], Suprapti et al. [15], and Setiyaning et al. [16] assessed the impact of probiotics supplementation on immune/inflammatory markers.

##### 3.4.5.1. Outcomes of Probiotic Supplementation Without Adjuncts

Jiang et al. [13] found no significant differences in the numbers of neutrophils, lymphocytes, monocytes, and eosinophils post probiotics supplementation. Moreover, high-dose probiotic supplementation was associated with significantly reduced TNF-α, IL-6, IL-10, and IL-12 levels compared with the control and low-dose groups. However, no significant change was observed in IFN-γ concentrations across the groups [13].

##### 3.4.5.2. Adjunctive Probiotic–Micronutrient Supplementation

Setiyaning et al. [16] found that post probiotics and zinc supplementation led to a significant increase in lymphocyte levels, whereas monocyte levels and neutrophil–lymphocyte ratio (NLR) were significantly decreased after probiotics supplementation.

Suprapti et al. [15] reported that in the probiotic group, plasma IFN-γ levels initially increased after one month of treatment. However, it showed a significant reduction by the end of 2 months compared with the baseline. Likewise, IL-12 levels peaked after one month but significantly declined by the second month.

#### 3.4.6. Systemic Metabolite Profile

Jiang et al. [13] reported metabolomic modulation in response to probiotic supplementation. They identified 32 common differential metabolites across the groups of nonprobiotic, low-dose, and high-dose probiotics. Among these, 11 metabolites showed significant changes, particularly in the high-dose group. Key upregulated metabolites included pyridoxamine, L-saccharopine, phosphatidylserine (PS), maresin 1 (MaR1), and several phosphatidylcholines (PC), while downregulated metabolites included phenylalanine, N-acetylmethionine, phosphatidylethanolamine (PE), and L-tryptophan. Findings of Jiang et al. [13] indicated that high-dose probiotic supplementation induces significant alterations in systemic metabolite profiles, particularly involving metabolites implicated in immune modulation and anti-inflammatory pathways.

### 3.5. Consideration of Key Modifiers on Studied Patient's Characteristics

We have reviewed the exclusion criteria and summarized them in Table 3. Most studies excluded participants with significant comorbidities such as cardiovascular disease, liver disease, malignancy, or severe mental illness, which reduces variability but may limit generalizability to high-risk populations [11–15]. Only one study [15] explicitly excluded patients with HIV, while others [11–14, 16] did not report HIV status. Diabetes was excluded in several studies [12–15]. Alcohol and tobacco use were largely not reported, making it difficult to assess their impact on outcomes. Prior probiotic use was excluded in some studies [11–14], reducing confounding. Importantly, none of the studies mentioned nutritional assessment or food habits, which are key determinants of gut microbiota composition and may significantly influence probiotic efficacy. Overall, these key modifiers may influence probiotic efficacy, and the variability in reporting highlights the need for future studies to clearly document these factors to better interpret outcomes across diverse patient groups.

## 4. Discussion

This scoping review is the first to comprehensively synthesize evidence on the role of probiotic supplementation in TB patients, examining its impact across gut microbiota composition, ADRs, immune modulation, and systemic metabolomic changes.

We observed that existing research on probiotics supplementation in TB focuses primarily on PTB populations, with limited studies exploring its role in MDR-PTB or EPTB patients. This limited evidence gap of probiotics uses in MDR-PTB and EPTB populations suggests a future scope for research.

Treatment of TB usually consists of a multidrug regimen. The requirement for multidrug regimens is linked to a higher occurrence of ADRs. These ADRs can range from mild to fatal. A significant ADR associated with one of the anti-TB medications, resulting in the cessation of that drug, is linked to various complications, including heightened morbidity and mortality rates. ADRs may vary from mild GI disturbances to severe hepatotoxicity, peripheral neuropathy, and dermatological ADR, among others [17]. Most ADRs are observed in the intensive phase compared to the continuation phase [18]. Dixon et al. [19] reported ATT-induced ADRs significantly contribute to patient-originated missed doses of ATT, with GI, hepatic, neurological, cutaneous, and psychiatric ADR being primarily associated [19]. Our review findings showed a precise dose-dependent response to probiotics supplementation of *Lactobacillus casei* with higher doses associated with reduction in both GI and hepatic ADRs during ATT in patients with PTB [12, 14]. Evidence shows that patients with EPTB have over twice the odds of experiencing ADRs compared with those with PTB [20]. This emphasizes the importance of expanding future research to evaluate the potential of probiotics supplementation in all types of TB. By alleviating common and distressing ADRs, probiotic supplementation may result in better treatment compliance, particularly among patients who intentionally miss doses due to discomfort without informing their healthcare providers.

Our scoping review found that none of the included studies [11–16] supplemented probiotics beyond the intensive phase of TB treatment, which indicates that current research is focused mainly on short-term to intermediate outcomes from adjunct probiotics supplementation. However, it is essential to note that TB treatment lasts a minimum of 6 months to 2 years [1]. Furthermore, post-TB sequelae are a challenge regarding the decline in patients' health-related quality of life (HRQoL), and lung functions [21]. The research on the impact of probiotics supplementation on TB patients' HRQoL is still lacking. Research evidence from interventional studies indicates the positive effects of probiotics supplementation in chronic conditions like type 2 diabetes mellitus [22] and Crohn's disease [23]. Therefore, in growing recognition of the long duration of ATT, ATT-induced ADRs, and post-TB sequelae, future research may explore the role of probiotics throughout the full treatment course and into the posttreatment phase.

We found research evidence [15, 16] on probiotics cosupplemented with Vitamin B1, B6, B12, and zinc during ATT, which improved inflammatory markers. A systematic review and meta-analysis by Cabrera Andrade et al. 2020 found that micronutrient cosupplementation with zinc and vitamin A enhanced sputum conversion rates [24]. Moreover, they found that zinc supplementation alone was associated with a significant increase in weight, body mass index (BMI), and hemoglobin at the second and sixth months of supplementation [24]. TB patients often suffer from micronutrient deficiencies such as zinc, selenium, iron, vitamin B12, vitamin D, and vitamin A [25–27]. These deficiencies can exacerbate the severity of TB and impair the body's ability to recover effectively. Therefore, probiotic cosupplementation with micronutrients may stabilize malnutrition, leading to better overall health and a more robust immune response.

TB disease and ATT are associated with gut microbiota dysbiosis, characterized by reduced diversity and depletion of beneficial commensals [2, 28]. Based on our review findings, probiotic supplementation contributed to partial restoration of gut microbiota balance, suppression of pathogenic traits in opportunistic microbes, and improved the metabolic activity of gut commensals symbionts [11, 12]. However, the extent of gut microbiota modulation remains limited, and current evidence available on probiotics intervention is limited to the intensive phase of TB treatment. Therefore, the long-term effects of probiotics intervention on gut microbiota modulation remain unidentified. This highlights the need for longitudinal studies to understand their efficacy and the mechanistic pathways through which probiotics modulate host-microbiota interactions in TB patients.

Another important consideration is the heterogeneity of probiotic strains and dosing regimens across studies. While some employed single-strain interventions such as *Lactobacillus casei*, others used multistrain formulations or cosupplementation with micronutrients, with variable doses and durations. Although one trial suggested a dose-dependent effect [14], the current evidence base is insufficient to establish optimal strain selection or dosing strategies. Future studies should, therefore, include longer-term evaluations and systematically investigate strain-specific and dose–response relationships to better inform clinical translation.

Our review findings showed that supplementation of probiotics reduced levels of ZO-1 protein, I-FABP, and LPS, especially when given in high doses (2 × 10^10^ CFU, two bottles of 100 mL per day) [12]. This suggests that the protective effect of probiotics supplementation on gut barrier function might be dependent. ZO-1 is a marker of tight junctions [29], I-FABP is a marker of enterocyte damage [30], and LPS is a marker of endotoxemia [31]. Dysbiosis of microbiota can compromise the epithelial barrier, resulting in a leaky gut and potentially triggering inflammatory responses [32]. Therefore, gut microbiota modulation by probiotic supplementation may better impact maintaining gut barrier integrity and reducing microbial translocation.

Review findings on immune/inflammatory markers suggest that probiotic supplementation may result in an immunomodulatory effect during the intensive phase of TB therapy [12, 15, 16]. *Mycobacterium tuberculosis* is recognized for its ability to interfere with host metabolic pathways, particularly those associated with inflammatory cytokine responses [33], which cause the alteration in systemic metabolites related to inflammatory cytokines [34]. Our review findings indicate that probiotics supplementation may partly revive metabolic homeostasis by impacting host immune-metabolic crosstalk, thereby highlighting a potential adjunctive role of probiotics in mitigating the immunometabolic dysregulation associated with active TB.

Acknowledging the potential risk of false positives in the clinical studies included in this review is essential. Due to relatively small sample sizes, multiple outcome measures, and heterogeneity in probiotic formulations and dosing regimens, some statistically significant findings may arise by chance rather than reflecting actual therapeutic effects. Additionally, the variability in study designs and measured endpoints further complicates the interpretation of the observed benefits of probiotics in TB patients. As this review did not include a formal risk of bias or statistical assessment for multiple comparisons, caution must be exercised when interpreting these early clinical findings. Larger, rigorously designed randomized controlled trials with standardized outcomes are necessary to confirm these findings and minimize the risk of type I errors.

A key limitation in the existing clinical evidence regarding probiotics as adjunct therapy for TB lies in its reliance on a relatively small number of studies. Especially, the randomized controlled trial by Lin et al. [14] and the associated post hoc analyses by Xiong et al. [12] and Otdushkina et al. [11] constitute a significant portion of the available data, which reflects that research in this area is still in its early stages, with most trials conducted among patients with PTB during the intensive phase of treatment. While these studies offer important insights into dose-related effects, their dominance limits the generalizability of the findings.

Only six studies were eligible for inclusion in this scoping review, reflecting the limited availability of clinical trials evaluating probiotics in TB patients. To our knowledge, no previous reviews have synthesized evidence in this area, indicating that research on probiotics as adjunct therapy in TB is still in its early stages. The small number of studies highlights important gaps, limited evaluation in MDR-TB and EPTB populations, heterogeneous probiotic strains, and under-reported patient characteristics.

In addition, it should be noted that all included studies administered probiotics only during the intensive phase of TB treatment, which is up to 2 months. This finding reflects a limitation of the existing evidence base. Since standard TB treatment regimens last at least 6 months and may extend up to 2 years for MDR-TB, there is a substantial gap in understanding the long-term efficacy, safety, and sustainability of probiotic supplementation. Addressing this gap through extended, multicenter clinical trials is essential to determine whether the observed short-term benefits persist across the full course of therapy and into the posttreatment period.

Future studies involving diverse TB populations, extended probiotic supplementation periods, and varied geographic settings are required. Expanding the scope and scale of research will be essential to fully understand the therapeutic potential of probiotics in improving treatment outcomes and mitigating ADRs in TB care.

## 5. Conclusions

This scoping review provides current evidence on how probiotics may support TB treatment, particularly during the intensive phase of ATT. Our findings imply that probiotics serve as a promising adjunct during the intensive phase of ATT by alleviating GI and hepatic ADRs. Furthermore, probiotic supplementation was found to modulate gut microbiota balance, improve gut barrier integrity, and modulate immune markers and cytokine-related systemic metabolites. There is no evidence of the long-term impact of probiotic use beyond the intensive phase, leaving a critical gap regarding their long-term impact throughout and after TB treatment. We recommend more robust, longitudinal studies to evaluate the full potential of probiotics in TB.

## Figures and Tables

**Table 1:** Characteristics of included studies.

Study ID [ref]	Country	Study design	Type of population	Probiotics composition	Outcome measured	Any other supplementation combined with probiotics	Number participant in probiotic group
Otdushkina et al. [11]	Russia	Prospective	MDR PTB	*Bifidobacterium bifidum* and *B. animalis* and *Lactobacillus casei*, *L. plantarum*, *L. delbrueckii* subsp. bulgaricus, *L. acidophilus*	21 days	None	30
Xiong et al. [12]	China	Post hoc analysis^∗^	PTB	Lactobacillus casei	8 weeks	None	220
Jiang et al. [13]	China	Post hoc analysis^∗^	PTB	*Lactobacillus casei*	8 weeks	None	32
Lin et al. [14]	China	Open RCT	PTB	*Lactobacillus casei*	8 weeks	None	275^∗∗^
Suprapti et al. [15]	Indonesia	pre–post test randomized control by time series	PTB and EPTB	*Lactobaccilus acidophilu*, *L. casei*, *L. rhamnosus*, *L. bulgaricus*, *Bifidobacterium breve*, *B. longum*, *Streptococcus thermophilus*	8 weeks	Vitamin B1, B6, B12	11
Setiyaning et al. [16]	Indonesia	Quasiexperimental with randomization	PTB	NA	4 weeks	Zinc	27

^∗^Xiong et al. [12] and Jiang et al. [13] are post hoc analyses derived from the same randomized controlled trial conducted by Lin et al. [14].

^∗∗^Lin et al. [14] included two probiotics intervention groups: one received a high dose (2 × 10^10^ CFU, two bottles of 100 mL per day) and the other a low dose (1 × 10^10^ CFU, one bottles of 100 mL per day) of *Lactobacillus casei.*

**Table 2:** Details of outcome measures.

Study ID [ref]	Gut microbiota composition (*n*)	Hepatic ADR/hepatological parameters (*n*)	Intestinal permeability (*n*)	Systemic metabolites	Immune/inflammatory markers (*n*)	Gastrointestinal ADR (*n*)
Otdushkina et al. [11]	30	✖	✖	✖	✖	✖
Xiong et al. [12]	220	220	220	✖	✖	✖
Jiang et al. [13]	✖	✖	✖	32	32	✖
Lin et al. [14]	✖	✖	✖	✖	✖	275
Suprapti et al. [15]	✖	✖	✖	✖	11	✖
Setiyaning et al. [16]	✖	✖	✖	✖	27	✖

*Note:* “*n*” implies the total number of participants in the probiotics group for which the particular outcomes were measures. “✖”: Particular outcome measure has not been performed.

Abbreviation: ADR, adverse drug reactions.

**Table 3:** Summary of key exclusion criteria in included studies.

Study ID [ref]	HIV exclusion	Diabetes exclusion	Alcohol/tobacco exclusion	Prior probiotics exclusion	Other key exclusions
Otdushkina et al. [11]	Not stated	Not stated	Not stated	Yes	Inflammatory and infectious diseases of the liver and intestines
Xiong et al. [12]	Not stated	Yes	Not stated	Yes	Pregnancy or lactation, combinations with other diseases including cardiovascular diseases, hematopoietic system diseases, gastrointestinal diseases, hepatitis B or C virus infection, fatty liver disease, malignancy or severe mental diseases; elevated liver enzymes
Jiang et al. [13]	Not stated	Yes	Not stated	Yes	Cardiovascular disease, hematological disease, gastrointestinal disease, liver malfunction, tumor, severe mental or psychological illness, or cognitive impairment
Lin et al. [14]	Not stated	Yes	Not stated	Yes	Cardiovascular diseases, hematopoietic system diseases, gastrointestinal diseases, hepatitis B virus infection, hepatitis C virus infection, fatty liver disease, elevated liver enzymes, receiving hepatotoxic drugs other than anti-TB therapy, diagnosed with malignancy, severe mental illness
Suprapti et al. [15]	Yes	Yes	Not stated	Not stated	Pregnancy, lactation, pneumonia, and current administration of corticosteroids or other immunosuppressants.
Setiyaning et al. [16]	Not stated	Not stated	Not stated	Not stated	Not stated

## Supporting Information

Preferred Reporting Items for Systematic reviews and Meta-Analyses extension for Scoping Reviews (PRISMA-ScR) Checklist.
